# Cognitive-coordination training: impact on sport-specific physical fitness and technical skill of adolescent basketball athletes

**DOI:** 10.3389/fpsyg.2025.1669608

**Published:** 2026-01-07

**Authors:** Qiner Li, Quan Fu, Longhui Li, Jingyi Wang

**Affiliations:** School of Kinesiology and Health, Capital University of Physical Education and Sports, Beijing, China

**Keywords:** cognitive-coordination, coordination ability training, athletes’ coordination ability, basketball training, cognitive-basketball

## Abstract

**Background:**

Coordination ability is crucial for athletic performance, particularly in basketball, which demands rapid decision-making under high cognitive load. However, few studies have examined systematic training that integrates cognitive and coordination components. This study investigated the effects of a 12-week Cognitive-Coordination Training (CCT) program on sport specific physical fitness and technical skills in adolescent basketball players.

**Methods:**

Thirty-two sub-elite basketball players were randomly assigned to an experimental group (EG, *n* = 16) or control group (CG, *n* = 16). The EG performed three 30-min CCT sessions weekly, embedded within the regular warm-up routine—replacing part but not all of it—while the CG followed standard training. Both groups completed pre- and post-tests of sport specific fitness (SR, AJT, DJR, SJTP) and technical skills (FDL, MPR, HSD).

**Results:**

No significant pre-intervention differences were found. After 12 weeks, the EG showed significantly greater improvements than the CG across all tests (*p* < 0.001). Within group analysis revealed significant gains in all measures for the EG (*p* < 0.001), while the CG showed smaller improvements in only four tests and in the overall mean score.

**Conclusion:**

The 12-week Cognitive-Coordination Training, embedded within the warm-up phase, effectively enhanced sport specific fitness and technical performance in sub-elite youth basketball players. By integrating cognitive processing with motor coordination, this approach offers a practical and theoretically grounded method for optimizing athletic performance and complements traditional basketball training models.

## 1 Introduction

### 1.1 Coordinative and cognitive demands in basketball

In the pursuit of elite athletic performance, the increasing complexity of technical and tactical actions in competitive sports necessitates athletes to develop higher levels of coordination ability (Boichuk et al., 2018). Basketball, as an open skill sport, exemplifies this requirement. It demands athletes to operate effectively within complex, fast-changing, and uncertain environments (Kamandulis et al., 2013; Koch and Krenn, 2021). During gameplay, players must rapidly identify relevant cues (often within 1–2 s) to distinguish between relevant and irrelevant stimuli and execute appropriate responses (Scanlan et al., 2014), all while performing technically challenging motor tasks such as ball control, passing, or shooting. This dynamic interplay illustrates that basketball performance is not merely a reflection of physical coordination, but also an embodiment of higher order cognitive functions integrated with motor control (Li et al., 2023).

Within the basketball context, athletes face the dual challenge of meeting both motor and cognitive demands (Schaefer and Scornaienchi, 2020). They must constantly monitor the positions and movements of opponents, teammates, and the ball, while swiftly repositioning themselves and selecting the most effective technique to exploit openings and score (Rahimi et al., 2022). Such tasks engage a range of cognitive capacities, including reaction ability, working memory, reasoning, and decision-making (Heisler et al., 2023). Moreover, the competitive nature of sport requires these cognitive processes to operate concurrently with the preparation and execution of complex motor responses (Schaefer and Scornaienchi, 2020).

To maintain stable performance amid frequent and rapid movement transitions, basketball athletes must achieve efficient integration between cognitive processing and motor execution (Lucia et al., 2021). This interaction reflects a dual task demand encompassing both cognitive and coordination capacities (Casanova et al., 2009), referred to in this study as Cognitive–Coordination. Such coordination enables athletes to maintain a dynamic balance among attentional allocation, motor control, and tactical judgment core components that underpin high level basketball performance (Kamandulis et al., 2013).

### 1.2 Theoretical foundation of cognitive-coordination training

According to Boichuk et al. (2018) and Botiaev (2010), coordination ability is a multifaceted composite capacity encompassing elements such as rhythm, balance, orientation, and reaction. Rigoli et al. (2012) further proposed that coordination ability and executive functions share overlapping cerebellar processing mechanisms, with neuroanatomical links between specific regions of the prefrontal cortex and cerebellum forming the structural basis for both coordination and executive control (Ridler et al., 2006). These findings highlight the intrinsic interaction between cognitive and motor systems. Similarly, Liu (2015) and subsequent studies (Gierczuk and Bujak, 2014; Jerzy et al., 2015; Bernstein, 2014) have shown that cognitive characteristics such as reaction ability, kinesthetic discrimination, motor adaptation, and spatiotemporal precision are closely associated with coordination ability.

**Figure 1:**
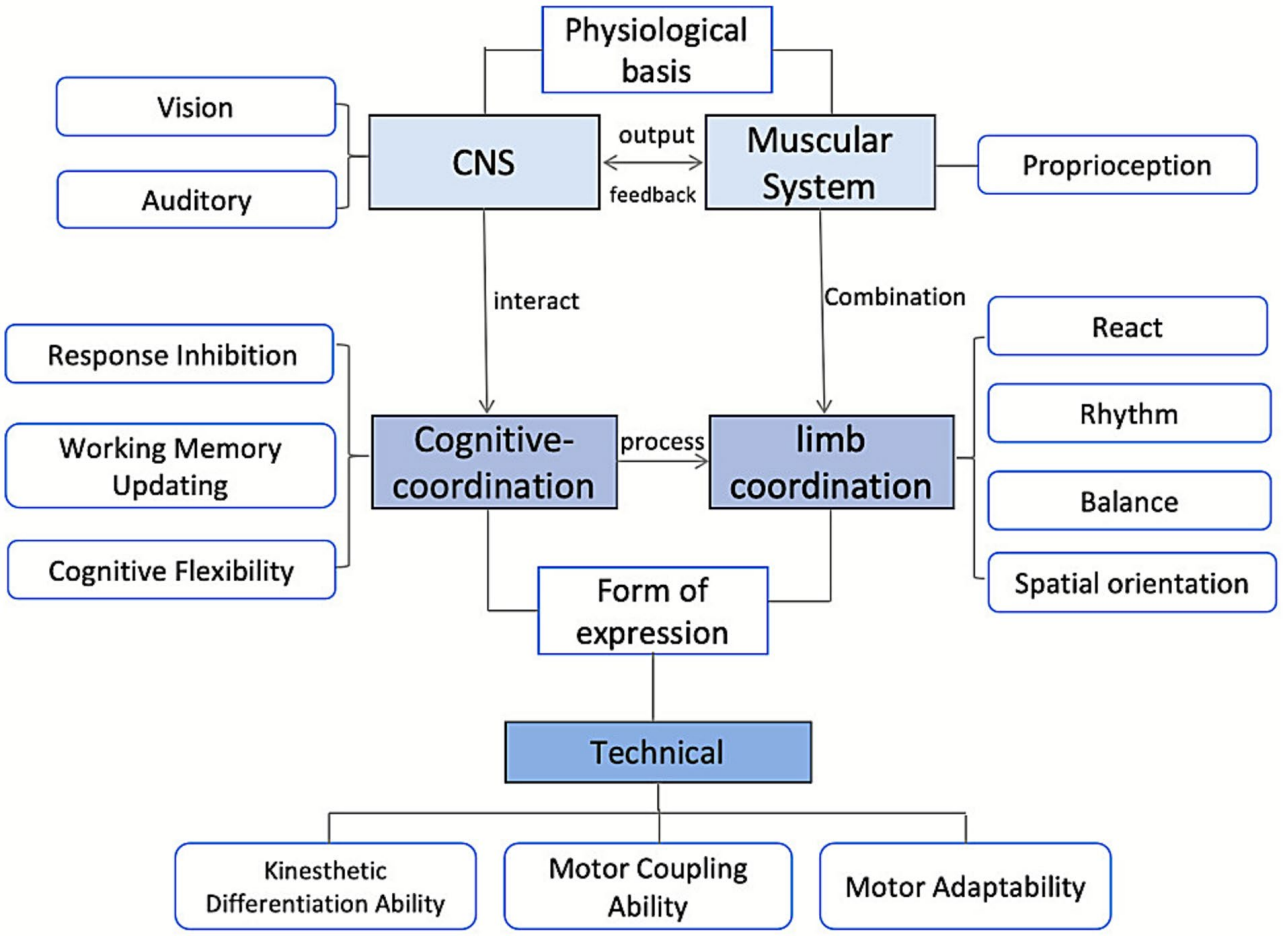
Structure of athletes’ coordination ability.

Building upon this theoretical foundation, the present study proposes a structural model of athletes’ coordination ability (Figure 1). This model positions cognitive coordination at its core, incorporating both basic reaction capacities and higher order executive functions,including inhibitory control, working memory updating, and cognitive flexibility (Diamond, 2013). Physical coordination comprises widely recognized motor components such as reaction, rhythm, balance, and spatial orientation. Within sport specific technical actions, proprioceptive differentiation, movement coupling, and adaptive control further reflect the integrative nature of coordination. This framework elucidates how cognitive processing and motor execution jointly contribute to overall coordination ability, providing a theoretical foundation for the development of cognitive coordination training strategies. By decomposing coordination into its cognitive and motor subcomponents, this model facilitates the systematic design and implementation of targeted training interventions.

Previous research has demonstrated that cognitive coordination training, an integrative dual task approach combining cognitive functions and motor coordination, can more effectively improve both coordination ability and cognitive performance in basketball players compared to traditional training methods (Zaichenko, 2023). Moreover, such training has been shown to enhance sport specific performance outcomes (Lucia et al., 2021). Therefore, the present study aims to further investigate the effects of a Cognitive-Coordination Training (CCT) program, grounded in the concept of cognitive motor integration, on various aspects of sport specific physical fitness and technical performance in adolescent basketball players. By integrating cognitive tasks with physical exercises, this program seeks to facilitate the development of executive functions such as planning, inhibition, and cognitive flexibility, thereby enhancing athletes’ motor control and technical execution in realistic competitive contexts. The study ultimately aims to provide a feasible and evidence based pathway for incorporating cognitive motor integration into youth basketball training.

## 2 Methods

### 2.1 Participants

This randomized controlled trial was conducted at the Basketball Training Center of the Capital University of Physical Education and Sports (CUPES), Beijing, China. A total of 32 adolescent basketball players (16 males and 16 females) from a municipal representative youth basketball team voluntarily participated in the study. Recruitment occurred between August and October 2025.

In the Chinese sports ranking system, “National Second-Level Athletes” is an official qualification indicating competitive level athletes at the sub-elite (provincial/regional) level. For international clarity, this classification is described as sub-elite basketball players throughout the manuscript. Inclusion criteria were as follows:

(1) Age between 15.0 and 15.9 years (±3 months);
(2) Officially certified as national second-level basketball athletes in China (corresponding to the sub-elite level);
(3) Participation in structured team based basketball training for ≥18 h per week during the previous 2 years;
(4) Absence of any musculoskeletal injuries limiting full participation in training within the past 3 months;
(5) Absence of neurocognitive disorders (e.g., attention deficit/hyperactivity disorder, learning disabilities) or visual impairments that could affect performance in reaction based tasks;
(6) Voluntary participation with written informed consent obtained from both athletes and their legal guardians.

Exclusion criteria included:

(1) Concurrent engagement in any other intervention targeting coordination or cognitive enhancement;
(2) Planned absences exceeding 20% of the intervention duration (e.g., external tournaments or school transfer);
(3) History of severe concussion within the past 6 months or lower limb surgery within the past year.

The study protocol was reviewed and approved by the Institutional Review Board of the Capital University of Physical Education and Sports (Approval ID: CUPES-IRB-2024A191). All procedures complied with the ethical standards of the Declaration of Helsinki. Legal guardians provided written informed consent, and each participant signed an individual assent form before enrollment.

Previous literature has shown that athletic expertise can attenuate gender differences in cognitive task performance (Alves et al., 2013; Ryan et al., 2004). Consultations with senior coaching experts also confirmed that gender differences in coordination ability are minimal among sub-elite adolescent athletes. Therefore, gender was not included as a primary analytical factor in this study. Instead, all analyses were conducted based on test scores and change values between pre- and post tests. Baseline comparisons showed no statistically significant differences between the experimental and control groups across all variables (*p* > 0.05), indicating good intergroup homogeneity before the intervention (Table 1).

**Table 1:** Baseline characteristics of participants (Mean ± SD).

Variable	Experimental group (EG, *n* = 16)	Control group (CG, n-16)	*p*-value
Gender (M/F)	8/8	8/8	–
Age (years)	15.4 ± 0.3	15.5 ± 0.4	0.62
Height (cm, male)	187.3 ± 3.2	185.1 ± 2.8	0.28
Height (cm, female)	172.6 ± 2.4	170.0 ± 2.5	0.25
Weight (kg, male)	73.5 ± 4.1	74.5 ± 4.3	0.58
Weight (kg, male)	58.0 ± 3.5	58.8 ± 3.8	0.63
Weekly training hours	18.6 ± 1.3	18.4 ± 1.5	0.77

No significant differences were observed between groups at baseline (*p* > 0.05), confirming homogeneity.

### 2.2 Experimental design and procedure

Following baseline assessments, all eligible participants (*N* = 32) were randomly assigned to the experimental group (EG, *n* = 16) or the control group (CG, *n* = 16) using sex stratified block randomization (block size = 4). The randomization sequence was computer generated by an independent statistician who was not involved in the intervention or assessments. Allocation concealment was ensured using sequentially numbered, opaque, sealed envelopes, which were opened by the project coordinator after baseline testing to reveal group assignments.

Both groups completed the same seven basketball-specific performance tests pre- and post-intervention, including four physical fitness indicators and three technical skill assessments. The CG followed a routine training program consisting of six weekly sessions (a total of 12 training units per microcycle). The EG followed the identical training schedule, except that the first 30 min of training on Mondays, Wednesdays, and Fridays were replaced with Cognitive-Coordination Training. Each CCT session was integrated into the warm up period and incorporated dual task elements designed to simultaneously challenge cognitive processing and motor coordination. All other aspects of technical, tactical, and physical training were kept fully consistent between groups.

Training load monitoring: to ensure comparable training load between groups, heart rate (HR) responses in all sessions were continuously monitored using Polar H10 sensors (Polar Electro Oy, Finland). As reported in a recent systematic review, male and female basketball players typically demonstrate HRmax values of 81.8–94.6% during active match play (Gutiérrez et al., 2024). Since the present intervention was administered during the warm-up phase, physical load was controlled within a moderate HR zone of 60–70%. In addition, the EG recorded behavioral performance metrics during cognitive motor sessions, including response accuracy (%) and error counts. These served as objective indicators of cognitive engagement and fatigue. The data were used to adjust subsequent training intensity to ensure that cognitive demands remained challenging yet tolerable, while maintaining comparable physical load between groups.

This dual-monitoring approach—combining physiological and behavioral indicator—allowed for precise regulation of both the physical and cognitive components of training load. It ensured that any observed performance improvements could be attributed to the integration of cognitive motor elements rather than differences in total workload or fatigue. Each training session was jointly supervised by the research team and certified coaches to ensure fidelity and consistency. Across the 12-week intervention, all athletes completed ≥90% of sessions, and no participant withdrew, sustained injuries, or deviated from the prescribed protocol.

**Figure 2:**
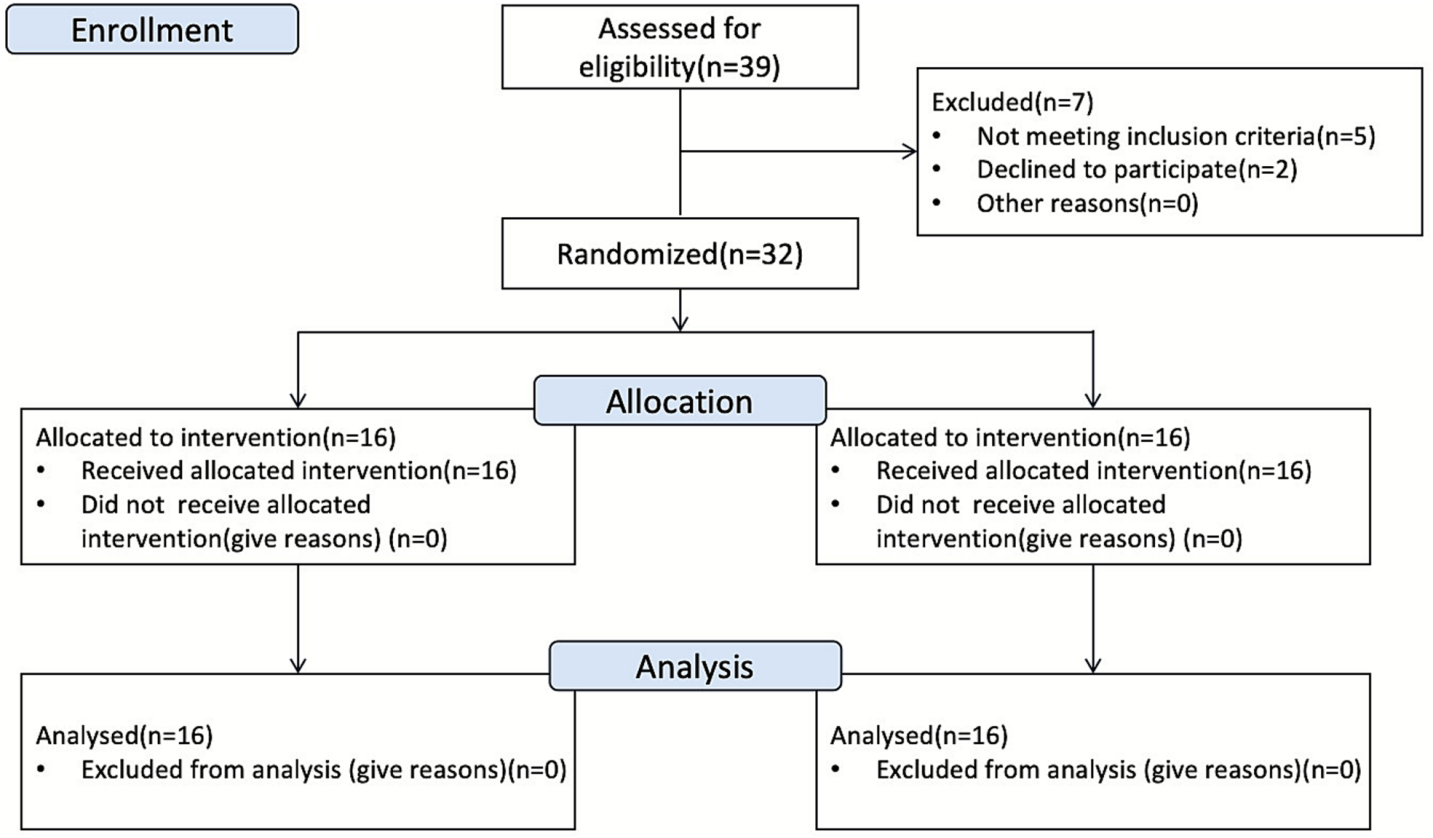
Experimental flowchart.

Post-intervention testing was conducted under identical conditions to baseline assessments, including the same time of day, environmental settings, and testing equipment. The overall study procedure (including recruitment, allocation, and assessments) is illustrated in Figure 2.

### 2.3 Cognitive coordination training (CCT) intervention

#### 2.3.1 Description of training methods

The CCT program was designed to enhance athletes’ integrated cognitive motor performance by simultaneously targeting fundamental coordination skills (balance, rhythm, and spatial orientation) and executive cognitive processes (inhibition, working memory, task switching, and decision-making). The intervention consisted of two progressive components: Basic Cognitive Coordination and Advanced Cognitive Coordination.

**Figure 3:**
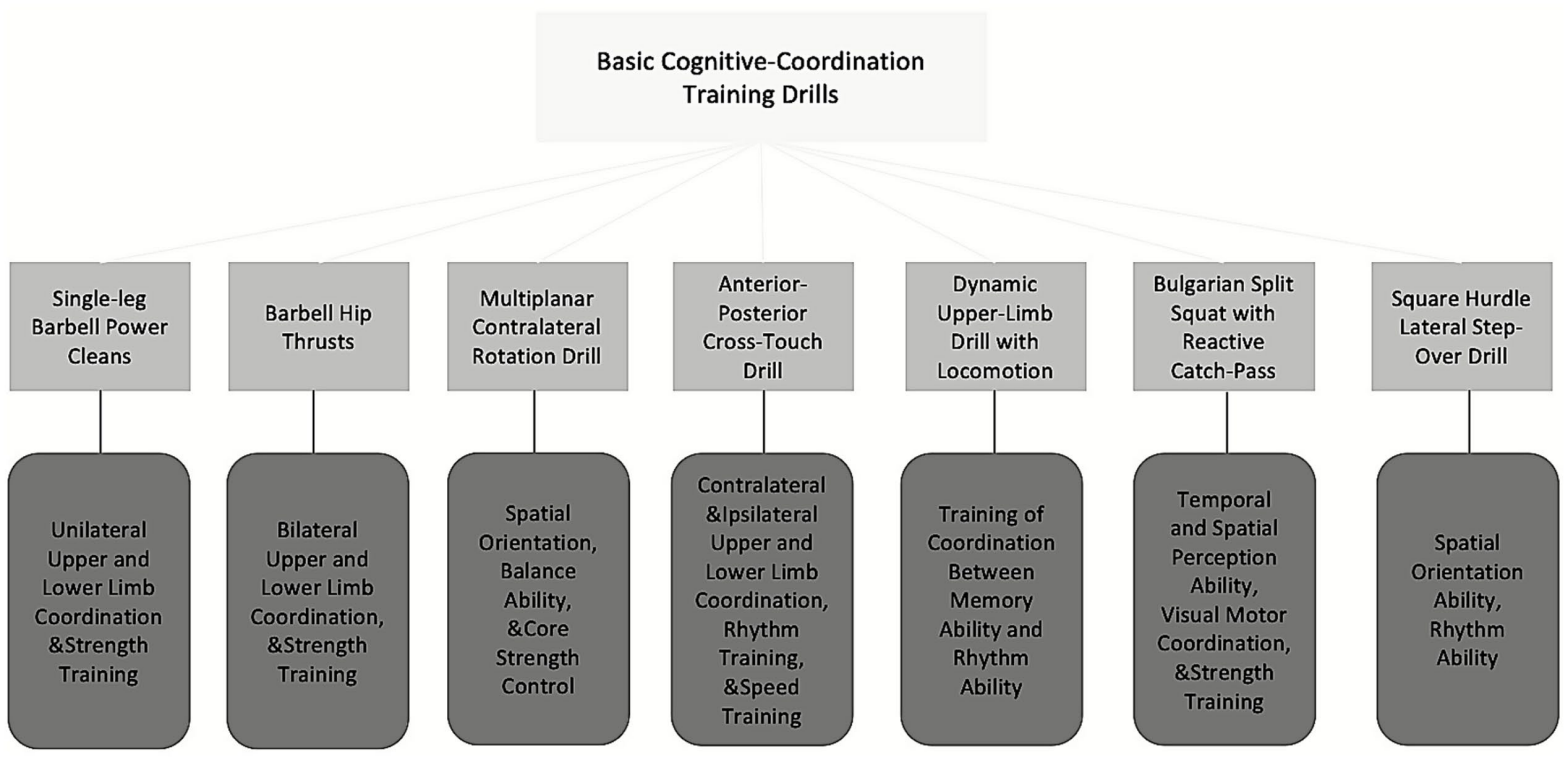
Basic cognitive coordination phase.

**Figure 4:**
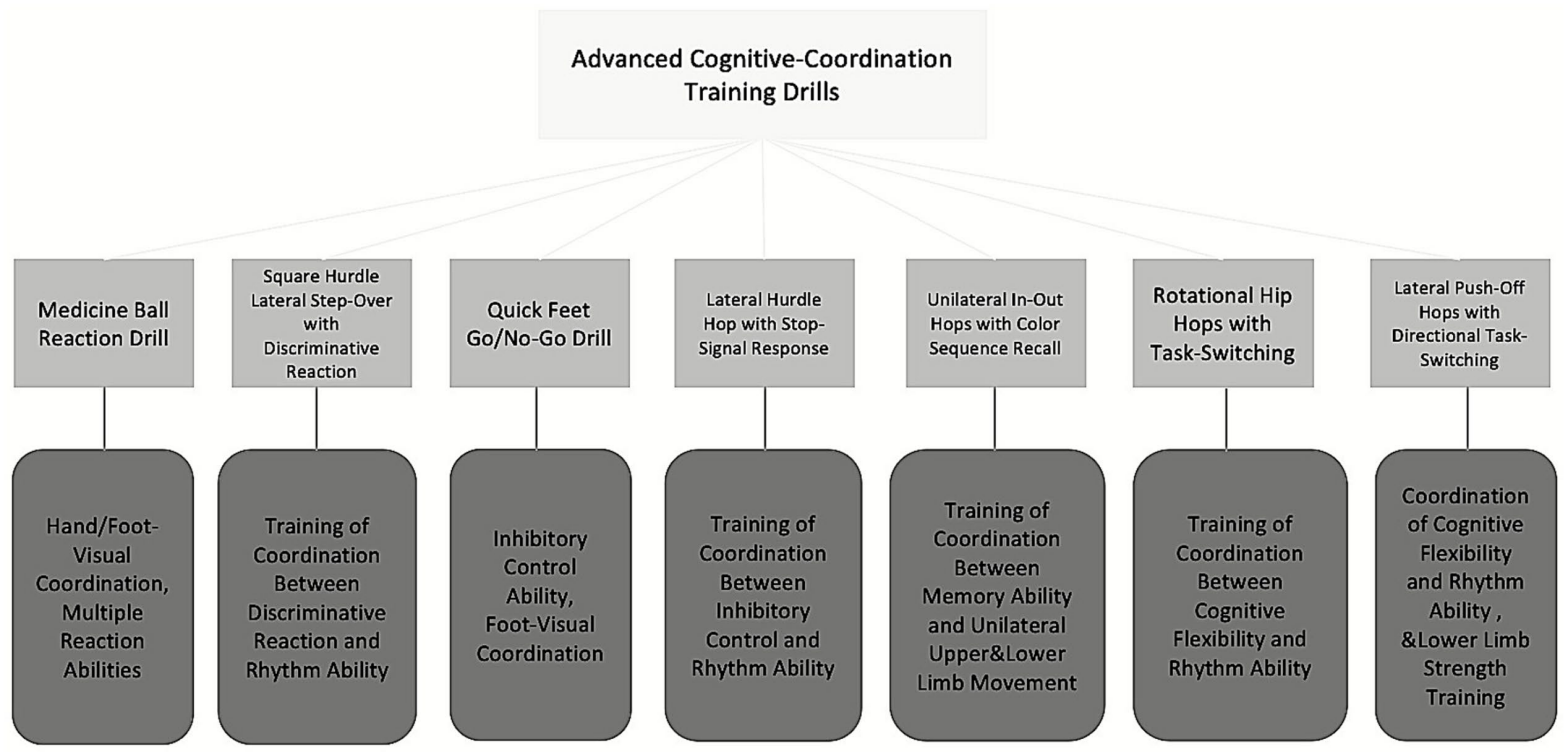
Advanced cognitive coordination phase.

The basic phase focused on developing fluency and stability in motor movements while introducing simple cognitive elements (Lopatenko et al., 2021). The advanced phase incorporated more complex cognitive challenges into basketball specific movement patterns. Detailed training methods and objectives are presented in Figures 3, 4, with complete protocols provided in Appendices 1, 2.

Unlike traditional coordination training, CCT tasks were designed with a strong emphasis on ecological validity. The cognitive motor dual task structure simulated perceptual and decision-making demands relevant to basketball. Light-based stimuli served not merely as generic coordination cues but as abstract representations of competitive game signals (Roca et al., 2018). For example, illuminated cues could signal direction changes, route memorization, or defensive reactions, thereby mimicking the dynamic and unpredictable information processing requirements encountered in real basketball situations (Albaladejo-García et al., 2023).

During training, athletes were required not only to respond to visual cues but also to interpret contextual meaning, integrate movement sequences, and continuously adjust motor output in real time. This approach aimed to improve perception–action coupling and decision-making efficiency in dynamic environments—both essential cognitive motor competencies in competitive basketball.

**Figure 5:**
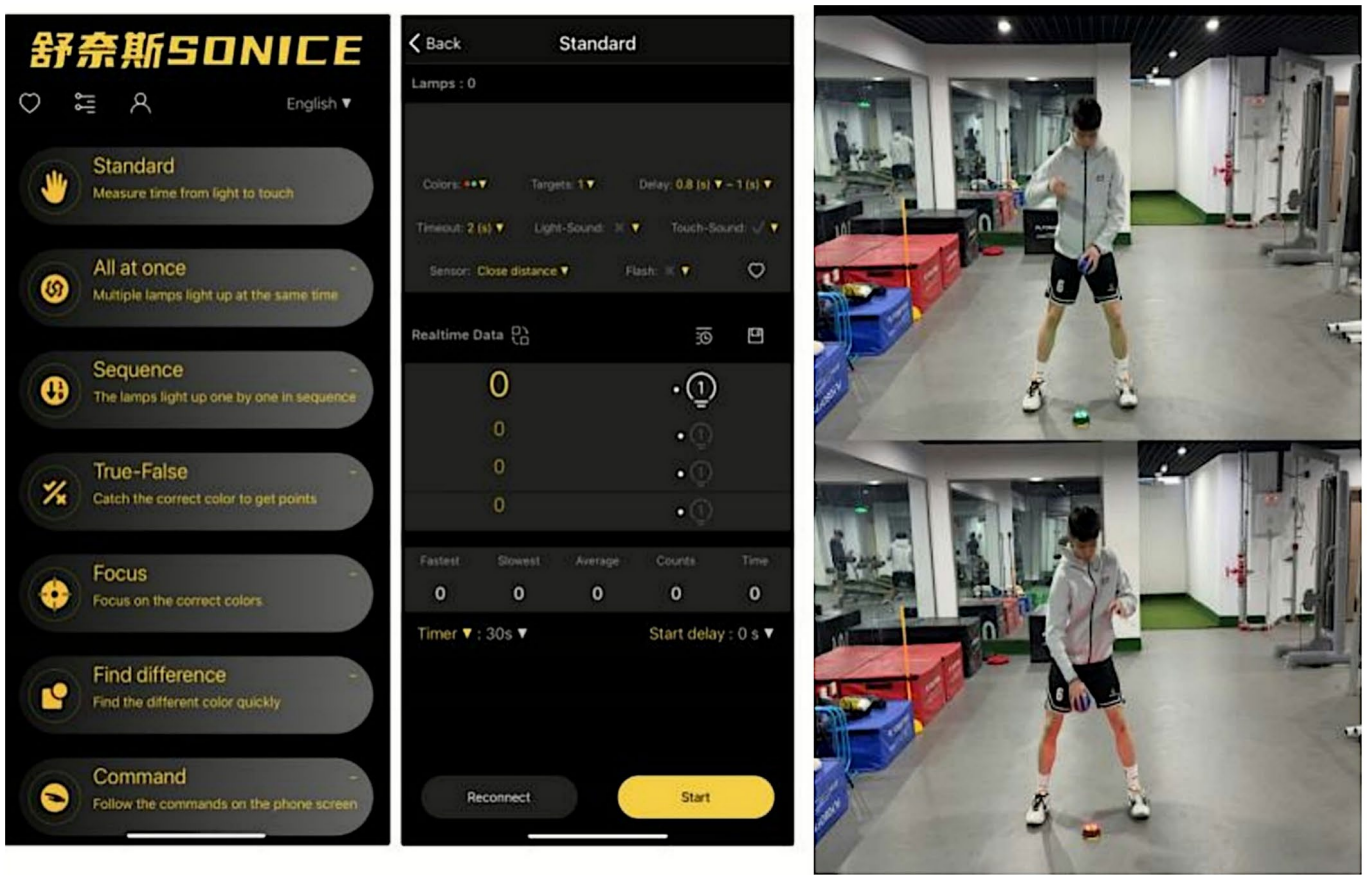
Soncic react training system.

The cognitive task components were implemented using the Soncie React training system (Figure 5; Patent No. ZL 2023 3005946.7). This system connects programmable reaction lights to a mobile application, enabling precise control of cue frequency, duration, and rule complexity. By continuously evaluating reaction time, accuracy, and error count throughout the session, the system provides real-time feedback and allows dynamic adjustment of cognitive load. This ensures that cognitive demands remain appropriately challenging while maintaining the intended progression of the training stimulus, consistent with contemporary recommendations for designing applied mental-workload contexts using interactive light-based devices.

#### 2.3.2 Quantification, progression, and monitoring of training load

In the CCT program, the design of the physical load was kept equivalent to that of the control group’s conventional fitness training. Resistance loads, repetitions, and sets were systematically matched to ensure comparable total energy expenditure and cardiovascular intensity across groups, thereby isolating cognitive load as the primary differentiating factor. Given that cognitive load reflects the demands imposed by cognitive tasks (Hassan et al., 2022; Duda, 2020), task difficulty was defined as the key determinant of cognitive training intensity, whereas task duration and frequency represented the cognitive training volume.

**Figure 6:**
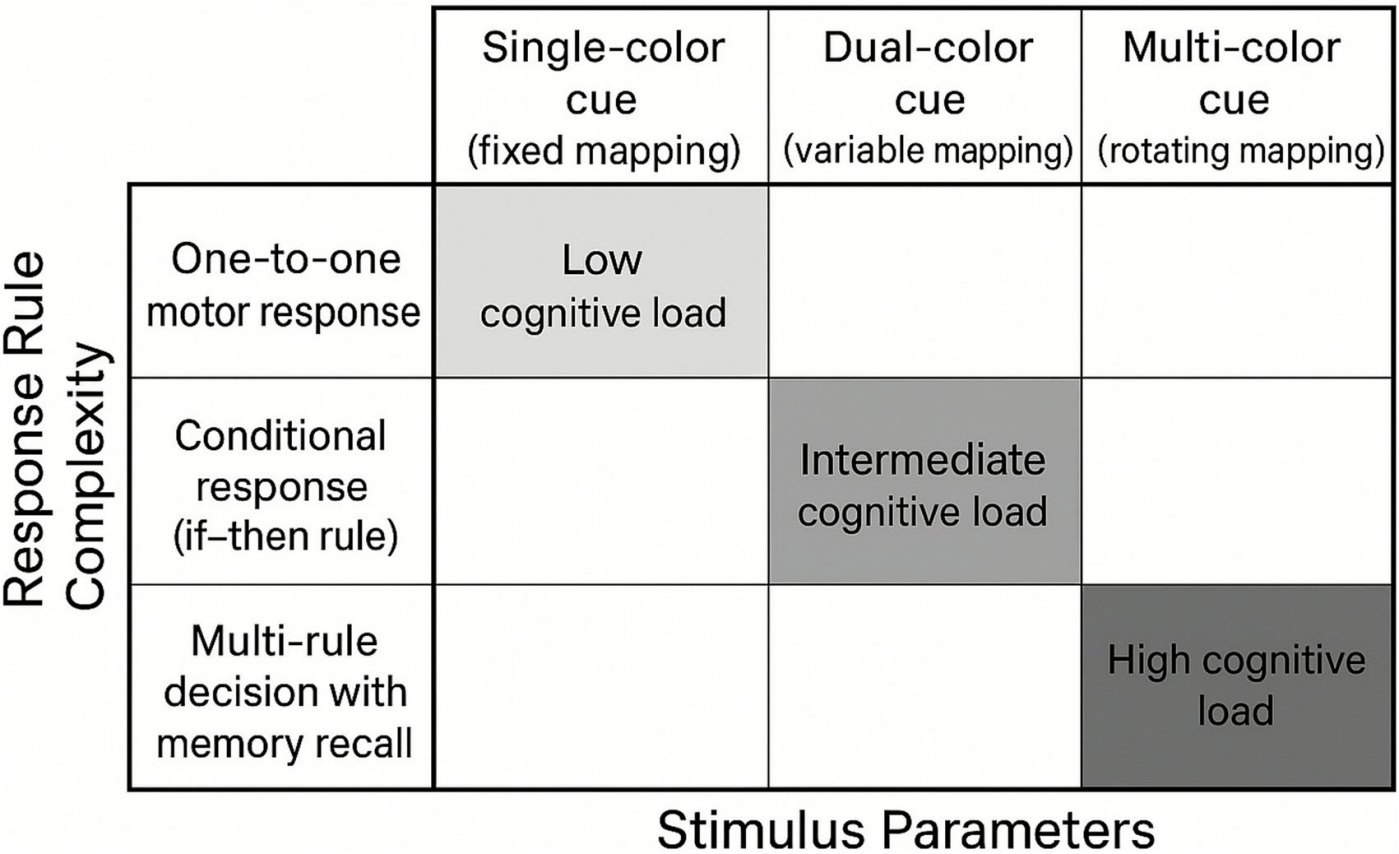
Cognitive difficulty levels in cognitive-coordination training.

Cognitive difficulty was operationalized through two components—stimulus parameters and response-rule complexity (Figure 6)—as previously described in cognitive control literature (e.g., Gutiérrez-Capote et al., 2025; Parrington et al., 2015; complementing earlier foundational work such as Dreisbach et al., 2007).

Stimulus parameters included three levels:

Single-color cue (fixed mapping): a consistent one-to-one stimulus response pairing (e.g., red → left step), emphasizing basic perception–action coupling.
Dual-color cue (variable mapping): two alternating colors linked to different motor responses, requiring inhibitory control and rule switching (e.g., red → left; blue → right).
Multi-color cue (rotating mapping): three or more colors presented in randomized sequences, demanding rapid updating, greater cognitive flexibility, and enhanced adaptability.

Response-rule complexity was also structured into three levels:

One-to-one motor response: A direct correspondence between stimulus and movement (simple response execution).
Conditional response (if–then rule): Context dependent mapping requiring selective attention (e.g., if red and top light → pass; if blue → dribble).
Multi-rule decisions with memory recall: Actions based on stored sequences or cue combinations (e.g., respond only to the third cue in a sequence), emphasizing working memory, inhibitory control, and higher-order decision-making.

The cognitive training load was manipulated by adjusting the task duration and the inter-stimulus interval (ISI) using the Sonice system. To maintain methodological transparency, cognitive load was categorized into three progressive levels—Simple, Moderate, and Advanced—based on quantifiable task parameters. Progression followed four dimensions:

(1) Increasing S–R mapping complexity: from fixed to variable to context-dependent mappings;
(2) Shortening ISI: from 3–4 s (simple) to 0.5–1 s (advanced);
(3) Increasing rule complexity: from simple reactions to tasks requiring inhibition of distractors and rule switching;
(4) Increasing concurrent elements: athletes performed sequences ranging from single to multiple actions, elevating working-memory demand.

**Figure 7:**
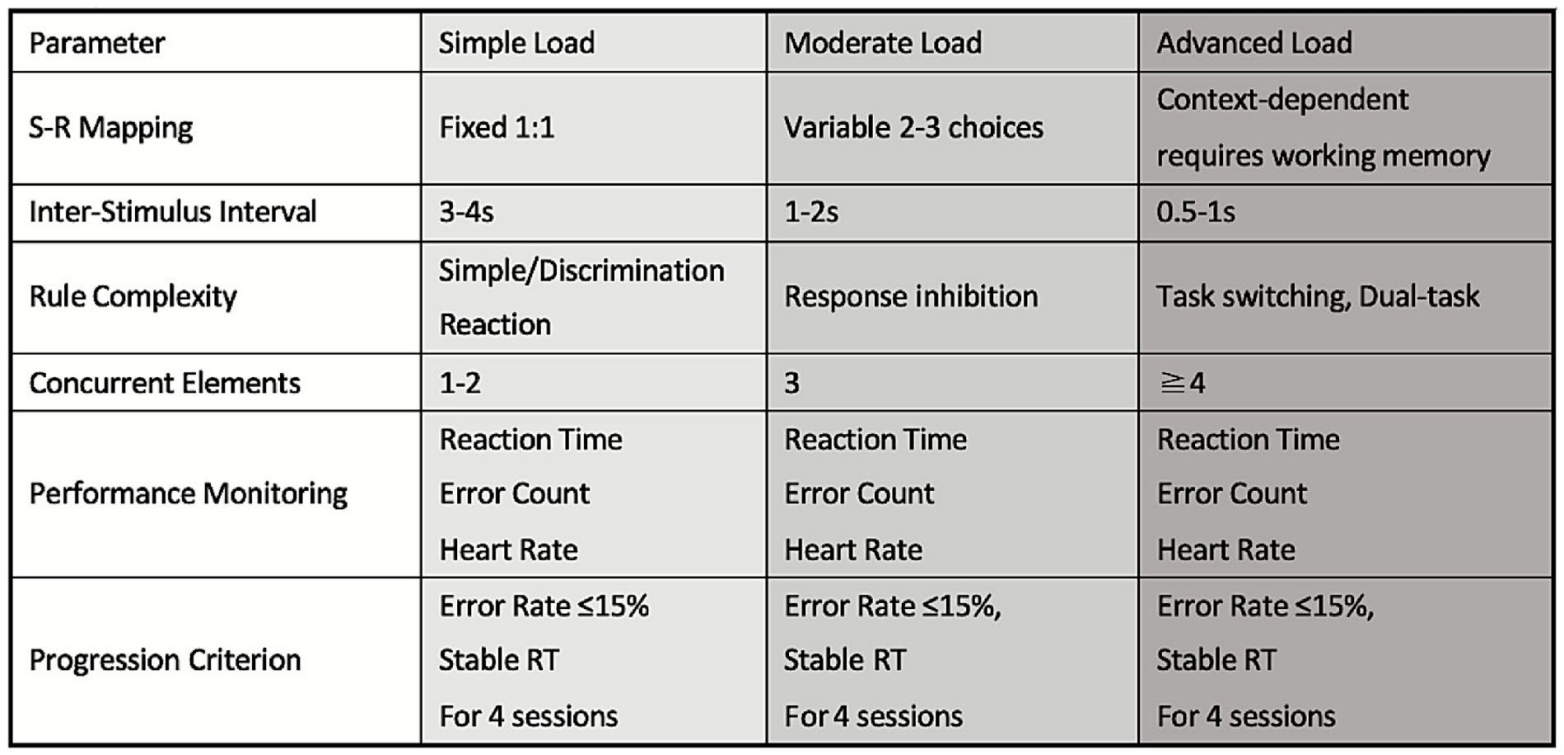
Cognitive load levels in cognitive-coordination training.

These criteria collectively defined the three-level cognitive load classification illustrated in Figure 7.

To minimize inter individual differences in baseline cognitive ability and fatigue tolerance, a pre-test calibration was conducted prior to the intervention. During training, the Sonice React system automatically adjusted cue timing and color frequency within predefined boundaries to maintain comparable challenge levels and reduce predictability. Functional difficulty was continuously monitored using two objective indicators:

(1) Task performance, with reaction times and error types (missed cues, incorrect responses) automatically recorded;
(2) Physiological responses, with HR continuously monitored as previously described.

To further ensure individualized yet standardized training progression, additional monitoring criteria were applied:

- Difficulty adjustment: if an athlete’s error rate for a specific action remained below 10% within a session, difficulty was increased in the subsequent session.
- Progression criteria: advancement from one cognitive load level to the next required an average error rate ≤15% across four consecutive sessions, accompanied by stable or improving reaction times. This ensured that nominal difficulty reflected equivalent functional challenge across individuals.
- Fatigue monitoring: a sudden increase in errors (e.g., >25% within a session) was treated as a behavioral marker of cognitive fatigue, prompting adjustments in rest intervals or instructional feedback.

Using this integrated, data-driven monitoring framework, cognitive load was treated as a quantifiable and modifiable experimental variable. Continuous assessment of both physiological and behavioral metrics enabled precise regulation of cognitive intensity across sessions and participants, ensuring consistent and reproducible measurement of combined physical and cognitive demands.

#### 2.3.3 Intervention phases

**Figure 8:**
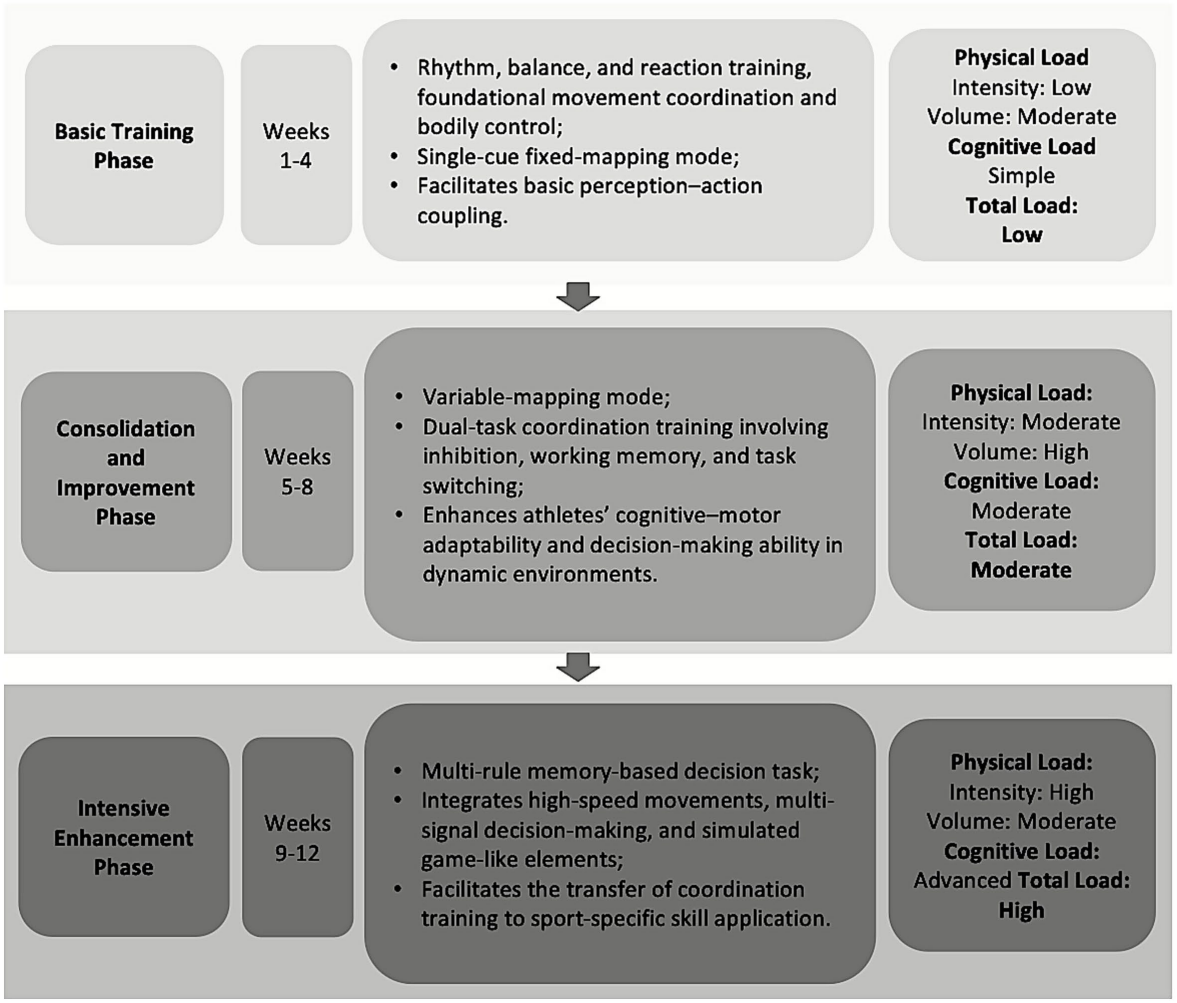
Cognitive-coordination training intervention phases.

The 12-week CCT intervention was structured into three progressive phases, each emphasizing different aspects of cognitive motor integration and corresponding to increasing levels of sport specific perceptual cognitive demands (Figure 8).

##### 2.3.3.1 Foundation phase (weeks 1–4)

This phase focused on developing fundamental motor coordination, including rhythm, balance, and reactive control ability. Basic movement tasks were combined with low-complexity cognitive stimuli to introduce the concept of dual engagement. A single cue fixed S–R mapping was used to minimize cognitive demands and facilitate familiarity with dual-task execution, thereby reinforcing basic perceptual–motor coupling.

##### 2.3.3.2 Consolidation phase (weeks 5–8)

This phase adopted variable mapping structures and incorporated dual task coordination challenges involving inhibition, working memory, and task switching. Cognitive cues and movement responses became less predictable, requiring athletes to dynamically distribute attention and adjust their actions under moderate cognitive load. Guided by continuous monitoring of heart rate and accuracy, both cognitive and physical loads were progressively increased while maintaining equivalence to the control group’s training demands. By elevating cognitive task difficulty, this phase aimed to strengthen athletes’ cognitive motor adaptability and decision-making in dynamic environments.

##### 2.3.3.3 Intensification phase (weeks 9–12)

This phase implemented multi-rule, memory-based decision tasks that simulated real game perceptual demands under time pressure. High-speed movements, multi signal decision requirements, and sport specific elements were integrated. For example, light stimuli were configured to represent spatiotemporal cues analogous to teammate or opponent movements, requiring athletes to execute complex action sequences rapidly and accurately while maintaining situational awareness under high cognitive motor load. By aligning task requirements with the perceptual decision context of actual basketball play, this phase enhanced the ecological validity and sport specific transfer of coordination training.

Overall, the CCT program systematically integrated cognitive and motor challenges within a sport specific and ecologically valid framework by progressively embedding decision-making and perceptual cues into basketball related movements (Boraczynski et al., 2019). The combination of physiological monitoring (heart rate), behavioral metrics (accuracy, error rate), and structured load progression ensured comparability of functional training load across groups, thereby isolating cognitive coordination ability as the primary differentiating factor. This approach was designed to ensure that cognitive load modulation translated into sport specific perceptual–motor skills rather than generalized coordination improvements, supporting its practical relevance in athletic training.

### 2.4 Testing procedures

This study adopted the standardized basketball performance testing protocol for athletes aged 15–16 years as outlined in the Chinese Youth Basketball Training and Teaching Syllabus (3rd Edition, 2012), compiled by the Youth Sports Department of the General Administration of Sport of China. This testing framework has been widely used in national level youth basketball evaluations for over a decade and is recognized for its validity, reliability, and strong ecological relevance to real game contexts. The test battery comprehensively evaluates players’ physical fitness, technical execution, and psychological readiness, providing a holistic assessment of basketball specific performance. Prior to data collection, all participants were familiarized with the testing procedures to minimize learning effects and ensure test reliability. The assessment included four basketball specific physical fitness tests and three basketball technical skill tests, described as follows:

(1) 6 × 5.8 m Shuttle Run (SR): this test assesses the ability to accelerate, decelerate, and change direction rapidly, which are essential for in game defensive and offensive transitions. Unlike linear sprinting in track events, basketball specific speed involves the athlete’s capacity to start explosively, stop efficiently, and transition directions under control (Gao, 2018). During each shuttle segment, athletes must coordinate abrupt stops, pivots, and push-offs in one continuous sequence, reflecting a high level of coordination and speed integration.
(2) Approach jump touch with double leg stop and two hand touch (AJT): this test evaluates lower limb power, footwork coordination, and kinetic chain efficiency. The drill requires athletes to link two preparatory steps into a smooth rhythm, ensuring a controlled heel to toe weight transfer and balanced braking prior to takeoff. The quality of movement is judged by the smoothness of the stop to jump transition and the vertical reach achieved.
(3) 60-Second double under jump rope test (DJR): this test primarily measures coordination, agility, and spatial rhythm control. Athletes must perform two rope rotations per jump with consistent timing and rhythm. The ability to sustain rapid, smooth repetitions demonstrates superior temporal coordination between upper and lower limbs as well as high neuromuscular synchronization.
(4) Standing jump turn with overhead two handed pass (SJTP): this task assesses whole body coordination, explosive power, core stability, and upper limb strength. Participants are required to calculate the timing of the ball’s bounce and execute an upward jump with a controlled mid air rotation before completing a two handed overhead pass. Proper execution demands large movement amplitudes, fluid transitions, and precise timing, engaging the core (abdominal and spinal musculature) and upper extremity kinetic chain. The test reflects an advanced integration of strength, balance, and spatial awareness.
(5) Full court dribble layup under pressure (FDL): this technical test measures the speed and control of dribbling during full-court transitions. Athletes must memorize a sequence of dribbling routes and execute them rapidly before performing a layup. If the layup is missed, they must immediately adjust for a rebound and follow up shot, testing response inhibition and rapid decision-making. The entire sequence emphasizes precision, stability, rhythm variation, and smooth transition from ball handling to finishing under pressure.
(6) Multi point passing/receiving circuit (MPR): this task evaluates ball control and decision-making while moving. Participants are instructed to recall specific movement routes and technical actions to be performed at each designated spot. The challenge lies in maintaining rhythmic pacing and seamless transitions between passes and movements. Successful performance requires accurate spatial timing, fluid sequencing, and technical precision during both locomotion and ball handling phases.
(7) Hexagon shuffle drill (HSD): this test assesses reaction speed, footwork agility, and coordination in non-dribbling movement. Athletes are required to memorize twelve footwork sequences and respond instantaneously to cues dictating start to stop transitions. Efficient execution depends on high step frequency, dynamic lower limb power, stable center of gravity, and properly directed upper body motion. The HSD represents a compound measure of cognitive reactivity and motor adaptability, both critical to defensive and transition play.

Detailed test procedures and scoring criteria are provided in Appendices 3, 4.

### 2.5 Data analysis

All testing data were entered into Microsoft Excel to establish the primary database and analyzed using SPSS Statistics (Version 26.0; IBM Corp., Armonk, NY, USA). Prior to statistical analysis, the Shapiro–Wilk test was conducted to assess data normality. The results indicated that the data did not meet the assumption of normal distribution; therefore, non parametric tests were applied.

Within-group pre–post comparisons were analyzed using the Wilcoxon signed rank test. Between-group comparisons (Experimental Group vs. Control Group) were conducted using the Mann–Whitney U test. The significance level was set at *p* ≤ 0.05. Corresponding effect sizes (r) were computed to quantify the magnitude of differences and assess the practical significance of findings. A power analysis was conducted using G*Power 3.1 software to verify that the sample size provided adequate statistical power (power≥0.80) for detecting meaningful effects.

## 3 Results

After the 12-week Cognitive-Coordination Training (CCT) intervention, both the experimental group (EG) and the control group (CG) demonstrated varying degrees of improvement across the seven basketball specific physical fitness and technical skill tests. As shown in Table 2, within-group comparisons revealed that the CG exhibited significant progress (*p* ≤ 0.05) in a limited number of items (including DJR, SJTP, FDL, and HSD) as well as in the Composite Score (CS). No significant changes were observed in the other measures. In contrast, the EG showed statistically significant improvements across all seven tests and in the total composite score (*p* < 0.001).

**Table 2:** Within-group comparison of test scores before and after intervention.

Group		SR	AJT	DJR	SJTP	FDL	MPR	HSD	CS
CG	Z	−1.769	−1.479	$-3.136^{*}$	$-3.219^{*}$	−0.921	$-2.945^{*}$	$-3.088^{*}$	$-3.387^{*}$
	P	0.077	0.139	$0.002^{*}$	$0.001^{*}$	0.357	$0.003^{*}$	$0.002^{*}$	<0.001
	Negative rank/Ties	2/1	3/3	2/1	0/3	4/6	1/0	0/4	1/0
EG	Z	$-3.521^{*}$	$-3.619^{*}$	$-4.018^{*}$	$-3.531^{*}$	$-3.517^{*}$	$-3.522^{*}$	$-3.520^{*}$	$-3.516^{*}$
	P	$<0.001^{*}$	$<0.001^{*}$	$<0.001^{*}$	$<0.001^{*}$	$<0.001^{*}$	$<0.001^{*}$	$<0.001^{*}$	$<0.001^{*}$
	Negative rank/Ties	0/0	0/0	0/0	0/0	0/0	0/0	0/0	0/0

1. Negative ranks = Post-intervention < Pre-intervention; 2. Ties = Post-intervention = Pre-intervention. 3. *Indicates statistically significant differences.

Between-group comparisons revealed that prior to the intervention, there were no significant differences between the two groups in any of the test items or in the overall composite score (*p* > 0.05), suggesting that the two groups were equivalent at baseline. However, after the 12-week intervention, the EG significantly outperformed the CG in all seven tests and in the composite score (*p* ≤ 0.05), with the most pronounced differences observed in the 6 × 5.8 m shuttle run and overall total score (*p* < 0.001) (Table 3), indicating a more comprehensive enhancement following the intervention.

**Table 3:** Between-group comparison of test scores before and after intervention.

Pre- and post- testing		SR	AJT	DJR	SJTP	FDL	MPR	HSD	CS
Pre-test	Z	−0.397	−0.396	−0.359	−0.510	−0.548	−0.510	−0.076	0.000
	P	0.696	0.692	0.720	0.616	0.583	0.610	0.940	1.000
Post-test	Z	$-3.528^{*}$	$-3.019^{*}$	$-2.967^{*}$	$-2.924^{*}$	$-3.086^{*}$	$-2.164^{*}$	$-2.098^{*}$	$-4.129^{*}$
	P	$<0.001^{*}$	$0.002^{*}$	$0.003^{*}$	$0.003^{*}$	$0.002^{*}$	$0.030^{*}$	$0.035^{*}$	$<0.001^{*}$

*Indicates statistically significant differences.

**Figure 9:**
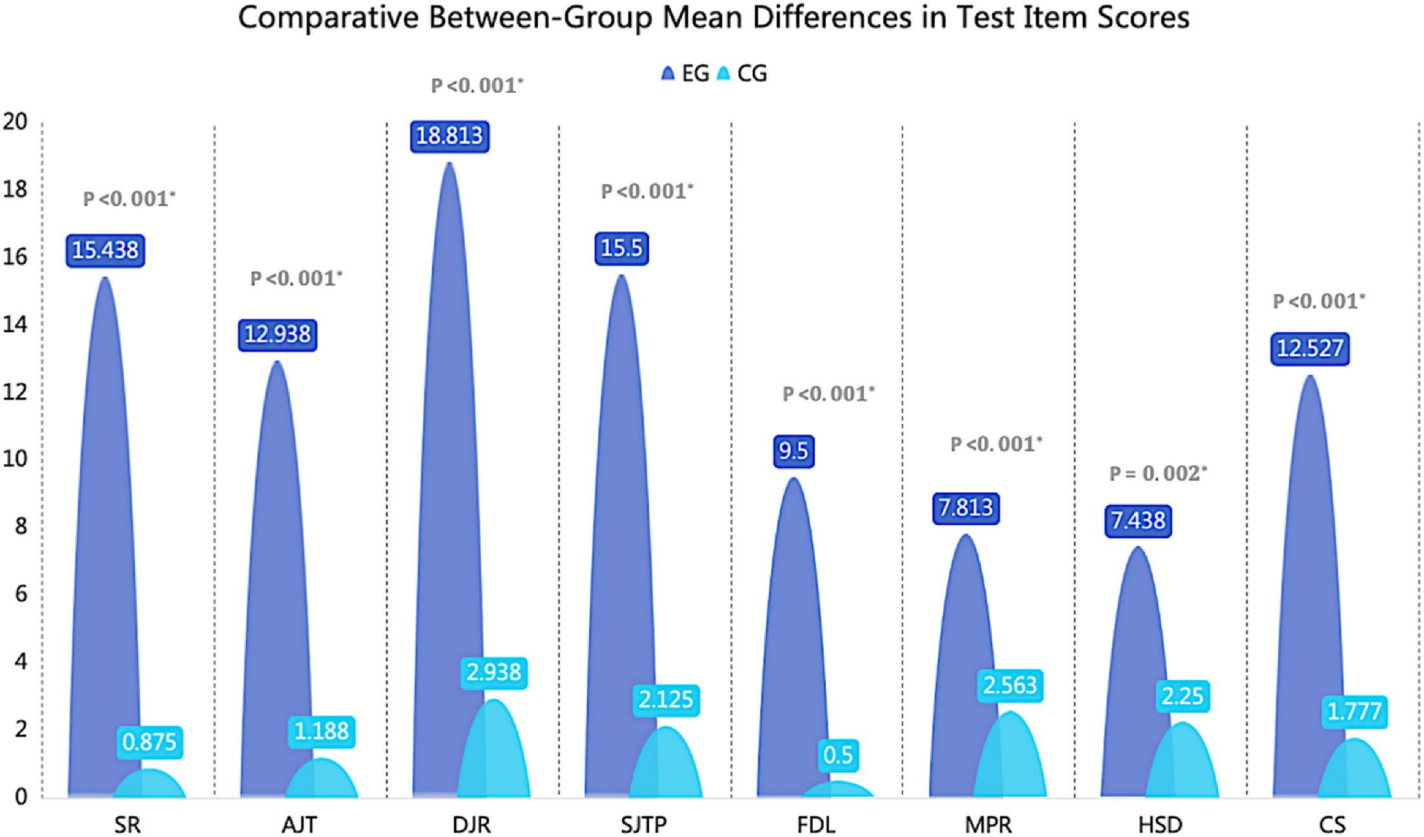
Between-group comparison of score differences before and after intervention.

To further examine the magnitude of improvement between the two groups, the changes in pre- and post-test scores were compared (Figure 9). The results showed that the EG exhibited an average increase of more than 12 points in basketball-specific physical fitness tests and an improvement of approximately 7–9 points in technical skill tests. In contrast, the CG showed only modest gains of about 0.5–3 points across all test items. Overall, the extent of improvement in every measure was significantly greater in the experimental group than in the control group (*p* ≤ 0.05), demonstrating the clear effectiveness of the cognitive-coordination training intervention in enhancing both physical and technical basketball performance.

Overall, these findings support the study hypothesis that the Cognitive-Coordination Training program, which grounded in the integration of cognitive processing and motor coordination, can substantially enhance sport specific physical fitness and technical skill in adolescent basketball players. This training approach strengthens the perception, decision and action coupling mechanism, enabling athletes to execute more precise and adaptive movements in complex, dynamic contexts than those achieved through traditional training methods.

## 4 Discussion

This study aimed to investigate the effects of a 12-week Cognitive-Coordination Training (CCT) intervention on sport specific physical fitness and technical performance in adolescent basketball athletes. The results showed that the experimental group (EG), which received CCT in addition to regular training, demonstrated significantly greater improvements than the control group (CG) across all seven test indicators, as well as in the composite performance score. These findings suggest that incorporating cognitive tasks into coordination training may provide additional performance benefits beyond those obtained through traditional training alone.

However, these results should be interpreted with caution. Although the improvements observed in the experimental group were more pronounced, the present study did not include direct measurements of functional cognitive load, neurophysiological responses, or in-game performance indicators. Therefore, the findings indicate a significant association between CCT and enhanced sport specific performance rather than establishing a direct causal relationship. The observed advantages may more likely reflect synergistic adaptations in cognitive motor integration, supporting the potential value of embedding cognitive demands within coordination training to promote improvements in sport specific performance.

The results of this study are consistent with previous evidence suggesting that chronic dual task or cognitive motor interventions can enhance athletes’ coordination abilities and decision-making performance (Herold et al., 2018; Moreira et al., 2021; Wu et al., 2024). Such training has been shown to facilitate the concurrent development of motor control and executive functions, including working memory, inhibitory control, and cognitive flexibility. Ramírez Lucas et al. (2025) likewise reported that 8 weeks of cognitive motor training effectively improved physical attributes (speed, strength, and agility), cognitive performance (working memory, planning, processing speed, and reaction time), technical skills (dribbling and short passing), and dual task performance in adolescent soccer players. The CCT approach employed in the present study represents a similar model, indicating that incorporating cognitive tasks such as inhibitory control, working memory, and rapid decision-making may serve as a multidimensional training stimulus that enhances athletes’ task efficiency and coordination performance. These effects may stem from strengthened perception–action coupling and more efficient allocation of attentional resources (Wilson et al., 2005; Raab, 2012; Vestberg et al., 2017).

In terms of physical performance, the experimental group exhibited the most pronounced improvements in the DJR and SJTP tests. Kamandulis et al. (2013) reported strong correlations between physical fitness measures (including the 20 m sprint, Illinois agility test, and countermovement jump) and coordination measures, indicating that coordination ability in basketball players is closely associated with speed, agility, and lower-limb power. The CCT program implemented in the present study emphasized high-speed, low-load rapid transitions, reactive movements, and multi limb synchronized coordination under dynamically changing light cues. This training approach may have enhanced athletes’ rhythm adaptation and neuromuscular control. These elements can improve interlimb force transmission efficiency and timing precision—essential for explosive and reactive movements in basketball (Ostojic et al., 2006)—thereby potentially explaining the superior performance observed in the experimental group.

Regarding technical performance, the experimental group reduced task completion times by approximately 1–3 s, whereas the control group improved by only 0.2–0.3 s. In elite sport settings, millisecond level differences can determine competition outcomes (Darby et al., 2014), and the performance gap between groups provides further support for the potential advantages of CCT. However, part of the improvement may also be attributable to natural development or familiarization with the tests. Considering that the study was conducted during the winter training period characterized by intensive training loads, maturation and routine training effects cannot be dismissed. Therefore, the observed improvements should be interpreted as supplementary enhancements provided by CCT on top of traditional training, rather than the sole contributing factor.

Repeated exposure to cognitive load during training may allow athletes to select and execute motor responses more efficiently. Theoretically, such training can enhance athletes’ speed of stimulus interpretation and accuracy in response selection (Raab, 2012; Vestberg et al., 2017). Consequently, the adaptive changes observed in the experimental group are more likely to reflect improved attentional resource allocation and faster stimulus discrimination, rather than direct neurophysiological restructuring. Previous findings from related interventions support this interpretation. For example, Hassan et al. (2022) reported a 23–31% improvement in visual reaction time following FITLIGHT training, a system conceptually similar to the Sonnic system used in this study. Likewise, Badau et al. (2022) found significant improvements in multiple reaction time indicators after cognitive motor training. Basketball performance heavily depends on rapid interpretation of dynamic visual stimuli (teammates, opponents, the ball) (Lockie et al., 2014). By pairing visual cues (light signals) with specific motor responses, the CCT protocol may have enhanced visual tracking and working memory capacities (Panfil, 2011; Wright et al., 2005), which may help explain why the experimental group demonstrated improved dribbling proficiency and movement efficiency under high pressure conditions.

From a theoretical perspective, the findings of this study align with contemporary views on embodied cognition and ecological dynamics within the sport science field (Araújo et al., 2019; Raab and Araújo, 2019). These frameworks posit that perceptual motor learning emerges through dynamic interactions among task constraints, environmental cues, and individual capabilities. Within the CCT framework, the manipulation of visual stimuli (cue color, response mappings, and decision rules) can be interpreted as a controlled modulation of environmental constraints, thereby facilitating adaptive exploration of perception–action coupling.

Although the present study did not include neurophysiological assessments, prior EEG research has shown that cognitive-motor dual-task training can enhance the Bereitschaftspotential and frontal negative shifts, reflecting improvements in top-down control and inhibitory efficiency (Shibasaki and Hallett, 2006; Lucia et al., 2021, 2023). Based on these findings, it is plausible that CCT induced adaptive changes in attentional regulation, motor planning, and perceptual anticipation. Such mechanisms could help explain why the experimental group demonstrated substantial gains in tasks requiring rapid response selection and coordination under time constraints. However, due to the absence of direct neural or cognitive load measures in the current study, these interpretations remain inferential rather than empirically confirmed. Future research should incorporate methods such as EEG or fNIRS to provide more direct evidence.

The observation that the control group also showed significant improvements—albeit to a lesser extent—across four tests and the total score suggests the presence of several potential confounding factors. First, the study was conducted during the winter training period, a phase typically characterized by high training volume and intensity. Second, participants were in adolescence, a period during which natural maturation can contribute to performance improvements. Similar patterns have been observed in studies examining cognitive-motor dual-task training in semi-elite youth basketball players, where the control group also exhibited small performance gains (approximately 0.1–0.3 s) in both basketball specific tasks and cognitive tests following the intervention. However, the experimental group still demonstrated substantially larger improvements overall (Lucia et al., 2021, 2023). These findings, along with the present results, indicate that performance enhancements may not be attributable solely to CCT. Instead, CCT appears to function more effectively as a complementary component to traditional training.

Future studies should therefore incorporate additional control conditions, such as multiple intervention groups exposed to different levels of cognitive coordination load (e.g., moderate vs. high), and include direct measurements of in-game performance indicators (e.g., shooting percentage, rebounds, offensive and defensive efficiency). Such approaches would help verify the extent to which cognitive coordination training transfers to real-world sport-specific performance.

## 5 Limitations and future directions

(1) Lack of objective cognitive or neurophysiological monitoring. This study employed heart rate and behavioral error counts as indirect indicators for load control. While these measures ensured comparable physical loads, they also implicitly acknowledged the presence of additional cognitive demands. The experimental group incorporated training tasks with higher cognitive requirements, which may have altered overall physiological or psychological stress levels. Future research should adopt multidimensional monitoring approaches—for example, integrating Borg’s 6–20 Rating of Perceived Exertion (RPE) with the Cognitive Rating of Perceived Exertion (CRPE), supplemented by reaction time and accuracy measures—to more precisely quantify the physical and cognitive loads inherent in dual-task training.
(2) Lack of real-game performance data. Although the light-based cues were designed to simulate typical perceptual decision scenarios in basketball (e.g., reacting to directional changes, identifying passing targets, or responding to defensive pressure), these tasks cannot fully reproduce the perceptual complexity of real competition. Thus, while the observed improvements likely reflect enhanced perceptual motor coordination under controlled conditions, future studies should examine whether these benefits transfer to in-game performance indicators such as shooting accuracy, defensive positioning, or turnover rate.
(3) Potential influence of confounding variables. Improvements observed in the control group suggest that unmeasured variables (e.g., maturation, baseline cognitive ability, or changes in routine training intensity) may have contributed to the outcomes. Future studies may employ multi-arm designs (e.g., physical-only, cognitive-only, varying cognitive load levels) and stratified baseline grouping to minimize confounding influences.
(4) Single level of intervention intensity. This study included one experimental group and one control group, without exploring the dose–response relationship across different cognitive coordination training loads. Future work may incorporate multiple CCT intensity groups within randomized controlled trials and include long-term follow-up to determine the durability of effects and identify optimal training dosages.
(5) Limited sample representativeness. Participants were sub-elite adolescent athletes, which limits the generalizability of the findings to adult or elite populations. Future multi-center, multi-sport studies with larger sample sizes are encouraged to enhance external validity.

## 6 Conclusion

Despite these limitations, the present study offers practical insights for training applications. As a complementary modality to conventional training, Cognitive-Coordination Training (CCT) may be strategically incorporated into warm up or skill acquisition phases. Coaches may consider integrating short bouts of dual task modules to allow athletes to practice coordination under cognitively demanding conditions that partially simulate competitive scenarios. Such an approach may enhance athletes’ adaptive responses, working memory, inhibitory control, and task-switching capability, thereby promoting more integrated perception–decision–action processes. At the same time, overall training load should be carefully balanced, and the complexity of cognitive tasks should be adjusted according to athletes’ developmental stages.

In summary, this study provides preliminary evidence that CCT can improve sport specific physical fitness and technical performance in adolescent basketball players. From an applied perspective, CCT appears to be a valuable supplemental training approach. However, given the absence of direct cognitive load monitoring, neurophysiological assessment, and in-game validation, the observed benefits should be interpreted as associative rather than causal. CCT represents a promising direction for integrated training, but its underlying mechanisms and boundary conditions warrant further empirical investigation. Future research incorporating multimodal load monitoring and competitive context performance analysis will help clarify how cognitive demands and coordination abilities interact within real-world athletic environments to shape performance outcomes.

## Data Availability

The original contributions presented in the study are included in the article/Supplementary material, further inquiries can be directed to the corresponding author.
