# Supplementary material for: Cognitive-coordination training: impact on sport-specific physical fitness and technical skill of adolescent basketball athletes
Source: Front Psychol. 2026 Jan 7;16:1669608. doi: 10.3389/fpsyg.2025.1669608 (PMC12819253; doi:10.3389/fpsyg.2025.1669608)
Supplement: Supplementary file 1 [file Supplementary_file_1.docx]

Attachment 1：Basic Cognitive-Coordination Training Drills​

| Name | Methods |
| --- | --- |
| 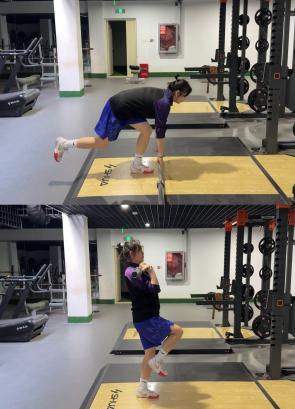  1.Single-leg Barbell Power Cleans​ | Stand vertically holding a 20kg barbell with arms fully extended.Maintain single-leg stance (left foot grounded),lift right leg backward until parallel to torso.Drive upward through triple extension (ankle-knee-hip),explosively shrug shoulders while pulling barbell vertically.Simultaneously rotate elbows forward under the bar,receive barbell on anterior deltoids with upright torso posture and achieve parallel alignment of right thigh-to-ground.  Sets:4; Reps per leg: 6-12;Load progression: Adjust based on individual force-velocity profile;Rest interval: 30-60s between sets |
| 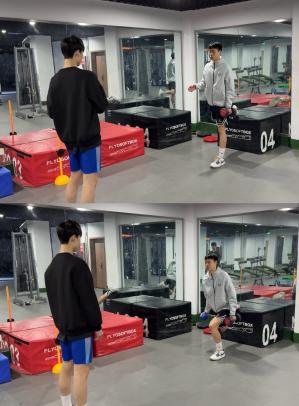  2.Bulgarian Split Squat with Reactive Catch-Pass​ | Athlete A:Performs squat holding 12-15kg dumbbell (single-arm contralateral load);  Athlete B: Throws medicine ball (2-4kg) from 3m distance.  Initiate eccentric phase (descending) → Partner releases med-ball;  Achieve full depth (hip-knee flexion ≥90°) → Catch ball with ipsilateral hand;  Immediate concentric drivetriple extension)→Return ball during ascent.  Sets: 4 ; Reps per leg: 8-12; Load adjustment: Based on reactive strength index (RSI); Rest: 30-60s between sets |
| 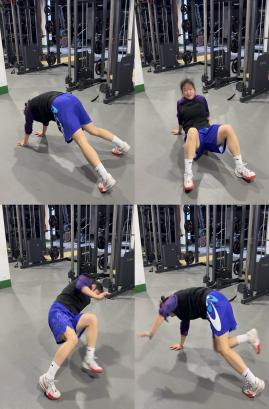  3.Multiplanar Contralateral Rotation Drill​ | Prone position with quadrupedal support (hands directly under shoulders, knees under hips);  Phase1:Lift contralateral limbs(left hand + right foot) simultaneously;  Phase2: Rotate torso through frontal plane to supine position;  Phase3: Maintain quadrupedal contact in supine orientation;  Phase4:Repeat limb lift → Rotate back to prone (360° completion);  Mirror sequence with opposite limbs (right hand + left foot).  Cognitive Load Progression:Add randomized auditory directional cues​(e.g., "Clockwise!" "Counter!")  Reps: 4–5 rotations per side; Sets: 4; Tempo: 2s eccentric→1s concentric |
| 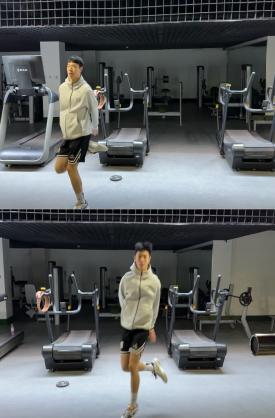  4.Anterior-  Posterior Cross-Touch Drill​ | Lift left knee toward chest (hip flexion ≥90°)→Right hand taps left foot；​  Lift right knee toward chest → Left hand taps right foot；​  Kick left foot toward right glute (knee flexion 110-130°) →Right hand taps left foot;​  Kick right foot toward left glute → Left hand taps right foot；  4 sets ×15–20m；  Tempo: 4-count cadence (anterior-posterior cycle/step)  Stage 1: Self-paced pattern acquisition  Stage 2: Auditory tempo synchronization  Stage 3: Reactive visual cueing |
| 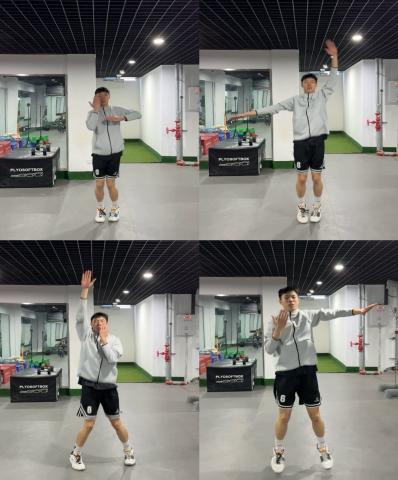  5.Dynamic Upper-  Limb Coordination Drill with Locomotion​ | Right Arm Kinematic Sequence​ (Sagittal → Frontal → Transverse Planes):  Phase 1: Shoulder flexion to 180° (palms outward);  Phase 2: Shoulder abduction to 90° (palms down);  Phase 3: Shoulder horizontal adduction + elbow flexion 90° (midline thoracic position);  Phase 4: Elbow flexion 130° + shoulder extension (vertical forearm position);  Left Arm Integration:Executes identical sequence with 1-beat phase delay;Bilateral synchronization maintained during hop-step progression.  Hop-step cadence: One movement phase per step;  Direction: Forward progression 15–20m;  Add stochastic visual cues(e.g., flashing directional indicators) |
| 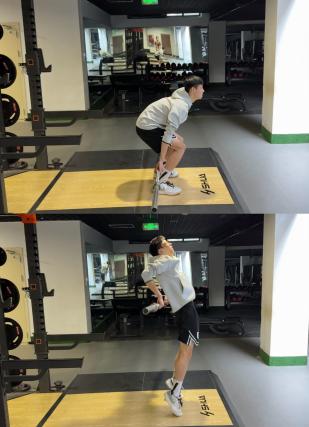  6.Barbell Hip Thrusts​ | Barbell positioned posterior to heels;Grip: Pronated (palms facing rear), shoulder-width;Initial stance: Half-squat position(knees ≈100-110°).  Concentric Phase:Drive through triple extension(ankle-knee-hip);  Elevate barbell along posterior kinetic chain  Terminate with:  ✓ Full hip hyperextension​ (anterior pelvic tilt)  ✓ Heels elevated (plantar flexion)  ✓ Barbell at gluteal fold level  Sets: 4; Reps: 12-14; Load adjustment: Based on hip extension torque capacity; Rest: 30-60s between sets |
| 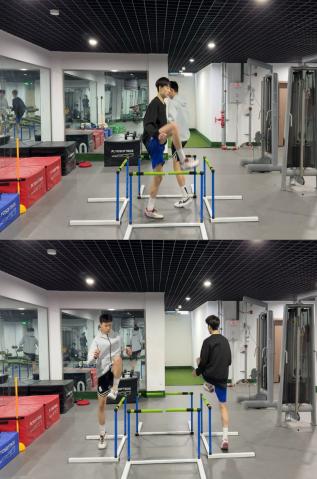  7.Square Hurdle Lateral Step-Over Drill​ | Four hurdles (height: 30-45cm) arranged in 1.5m × 1.5m square；Athletes positioned at diagonally opposite corners.Movement Sequence per Side:  Step 1: Lead leg (ipsilateral) lateral step into hurdle square;  Step 2: Trail leg lateral step into square → bilateral stance inside;​  Step 3: Lead leg lateral step out to adjacent side;  Step 4: Trail leg lateral step out → complete perimeter transition.  Tempo: Continuous flow at 3-4 cycles/second;  Duration: 30-45s continuous movement per set;  Directional Pattern: Clockwise → counterclockwise alternation;  Add stochastic auditory cues (e.g., "Switch!" "Reverse!"); Execute directional change within two step cycles.​ |

Attachment 2： Advanced Cognitive-Coordination Training Drills​

| Name | Methods |
| --- | --- |
| 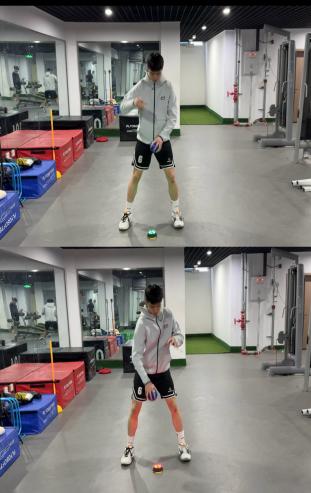  1.Medicine Ball Selective Reaction Drill​ | Face reaction light system(e.g., Soncie React) at 1.5m distance，hold medicine ball (4-6kg) with pronated grip (palms down).  Primary Task:  Continuously alternate ball snatches using finger flexion recruitment;  Maintain ball clearance height: 20-30cm above ground.  Cognitive Task:  Green light: Deactivate target with foot strike within 500ms;  Red light: Maintain ball snatch rhythm.  Neuromotor Constraints:Upper/lower limb dissociation required;  Zero palmar support (finger-tip control only);  Continuous motion during signal response  Duration: 30s/set × 4 sets;  Stimulus interval: Adjustable 0.3-2.5s (neurocognitive load progression) |
| 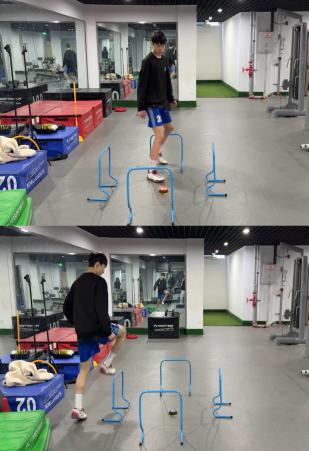  2.Square Hurdle Lateral Step-Over with Discriminative Reaction​ | Four low hurdles (30cm height) forming 1.5m × 1.5m square;  Central *Soncie React* light with color-coded directional mapping:  Red= Frontal hurdle (relative to recording camera); Yellow= Right-side hurdle;  Blue= Rear hurdle; Green= Left-side hurdle.  Neurocognitive Task:  Execute standard lateral step-over sequence；  Upon light activation: Jump designated hurdle within 800ms；  Maintain continuous movement during decision-execution cycle.  Visual stimulus interval: Adjustable 1.2-2.0s (cognitive load progression)  Movement precision: Foot clearance ≤5cm above hurdle crown;  Duration: 45s/set × 4 sets; Error tolerance: ≤2 missed reactions/set. |
| 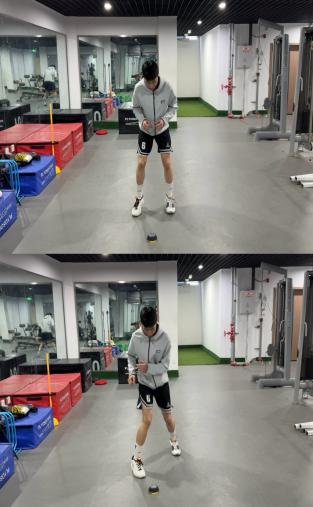  3.Quick Feet Go/No-Go Drill​ | Perform in-place quick feet facing reaction light system (e.g., DynaLight)；  Stance width: Shoulder-to-hip alignment；  Stimulus-Response Mapping:  Red light: Execute forward-backward hop → Deactivate light within 500ms  Green light: Perform lateral hop (frontal plane) → Deactivate light  Blue light: Maintain quick feet rhythm (inhibitory control)  Neuromotor Constraints:  Continuous plantar contact during baseline quick feet；  Full flight phase during hops (≥10cm vertical displacement)；  Zero upper-body compensatory motion.  Duration: 30s/set × 4 sets  Stimulus interval: Adjustable 0.3-2.5s (neurocognitive load progression)  Error tolerance: ≤1 failed inhibition/set |
| 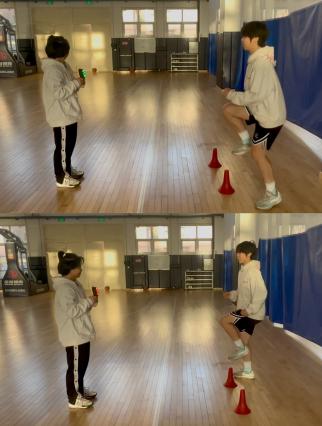  4.Lateral Hurdle  Hop with Stop-Signal Response​ | Initial position: Outside left hurdle (right foot lead)  Movement-Cognition Integration:  Green light: Execute continuous lateral hops over hurdle (left-right/right-left)；  Red light: Immediate movement cessation until light extinguishment；  Signal reactivation: Resume hopping within 300ms of light off.  Neuromotor Precision:  Hurdle clearance: ≥15cm vertical displacement；  Plantar contact time: ≤200ms during transitions；  45s/set × 4 sets;Stimulus interval: Adjustable 1.0-2.5s (cognitive load progression);  Error tolerance: ≤2 failed stops/set. |
| 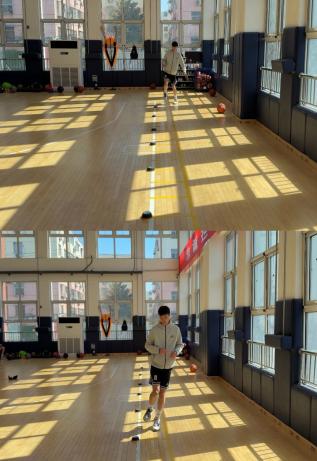  5.Unilateral In-Out Hops with Color Sequence Recall​ | Reaction lights sequentially placed along ladder’s far side；  Start position: Outside ladder (stance leg proximal to lights)  Movement-Cognition Integration:  Stance leg: Execute unilateral hops (in → out per rung)  Free leg: Deactivate lights in sequence during flight phase  Working memory: Verbally recall color sequence post-completion  Contralateral limb dissociation；Visuospatial working memory encoding；  Sequential processing under time constraints.​  Speed: Reduce light interval (1.2s→0.6s);  Complexity: Increase lights (4 →8 units);  Memory load: Lengthen sequence (3→ 7 colors). |
| 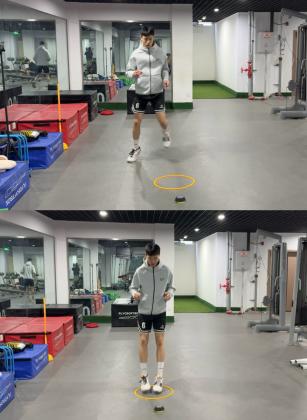  6.Rotational Hip Hops with Task-  Switching​ | Baseline Movement:Continuous in-place rotational hip hops；  1m facing obstacle ring (30cm diameter)  Green light: Execute lateral mini-hops(left-right/right-left) → Resume baseline  Red light: Double-leg jump into ring;Immediate ring exit;Backward hop to start position; Resume rotational hops  Neurocognitive Demands:  Task-switching efficiency(cognitive flexibility);  Spatiotemporal recalibration during transitions;  30s/set × 4 sets; Stimulus interval: Adjustable 1.0-2.0s (cognitive load progression)  Transition precision: ≤500ms signal-response latency. |
| 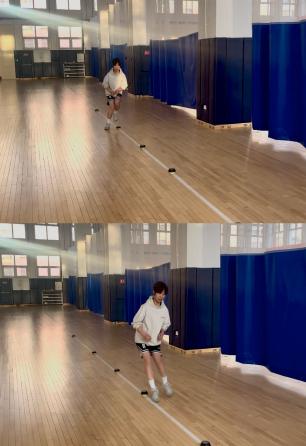  7.Lateral Push-Off  Hops with Directional Task-  Switching​ | Soncie React lights spaced 3-5m apart along 28m court baseline.  Basketball baseline facing first light.  Alternating lateral push-off hops advancing downcourt:  ✓ 45° torso lean toward movement direction;  ✓ Triple extension impulse ≥2.1×BW.  Cognitive Task-Switching:  Red light: Clockwise shuffle circle (1.5m diameter) around light;  Green light: Counterclockwise shuffle circle;  Resume lateral hops post-circle within 2 steps.  Distance: Full court (28m); Sets: 4;  Stimulus interval: Randomized 2.0-3.5s;  Circle precision: ≤0.5s deviation from perfect circumference. |

Attachment 3：Tests for basketball-specific physical fitness

| Name | Methods |
| --- | --- |
| 1.6 × 5.8m Shuttle Run | Athletes start from a stationary position behind the baseline under the basket, sprint to the free-throw line, execute a jump stop followed by a pivot turn, and return to baseline-this sequence constitutes one repetition. The test requires completing three full shuttles (baseline → free-throw line → baseline) at maximal speed. Each athlete performs two trials, with the better result recorded. |
| 2.Approach jump touch (two-foot landing, dual-arm reach)​ | Athletes perform a running start approach within the restricted area vicinity, executing a one-step takeoff followed by a two-foot jump stop, then explosively jump upward to touch the apex of the Vertec measuring device with both hands. Two trials are conducted, with the highest reach recorded as the final score. |
| 3.60-Second Double-Under  Jump Rope Test​​ | Athletes begin in the standardized starting position with the jump rope held behind the heels. Each valid repetition consists of one vertical jump with simultaneous double rotation of the rope passing completely underfoot.Participants maintain bilateral foot contact during takeoff/landing while executing coordinated arm swings. The test records the maximal number of consecutive double-unders completed in 60 seconds, with two trials performed and the highest count recorded. |
| 4.Standing jump turn with overhead two-handed pass​ | Starting from a stationary position behind the baseline facing away from the court, athletes execute a vertical jump with simultaneous 180-degree rotation, releasing the ball overhead with two hands while airborne. The pass must be completed before landing, projecting the ball toward the opposite end. The horizontal distance from the baseline to the ball's first contact point is measured. Two trials are performed, with the longer valid distance recorded. |

Attachment 4：Tests for basketball-specific technical skill

| Name | Methods |
| --- | --- |
| 1.Full-court dribble layup under pressure​ | From a stationary start behind the baseline, athletes:  Phase 1 (Right-hand):Dribble full-court using right hand;Executesequential moves: crossover→spin move→behind-back→between-legs;Finish with right-hand driving layup.Phase 2 (Transition):Secure rebound after made/missed shot;Immediately dribble back with right hand to half-court;  Execute right-hand driving layup at starting end.Phase 3 (Left-hand):Repeat full-court sequence with left hand；Perform all directional moves mirrored;Finish with left-hand layup.Scoring Protocol:Mandatory putback after any miss;Penalty: -2 seconds per missed putback，Max penalty: -5 seconds.Total time recorded from first dribble to last basket contact. Two trials performed with 5-min recovery, best score recorded.  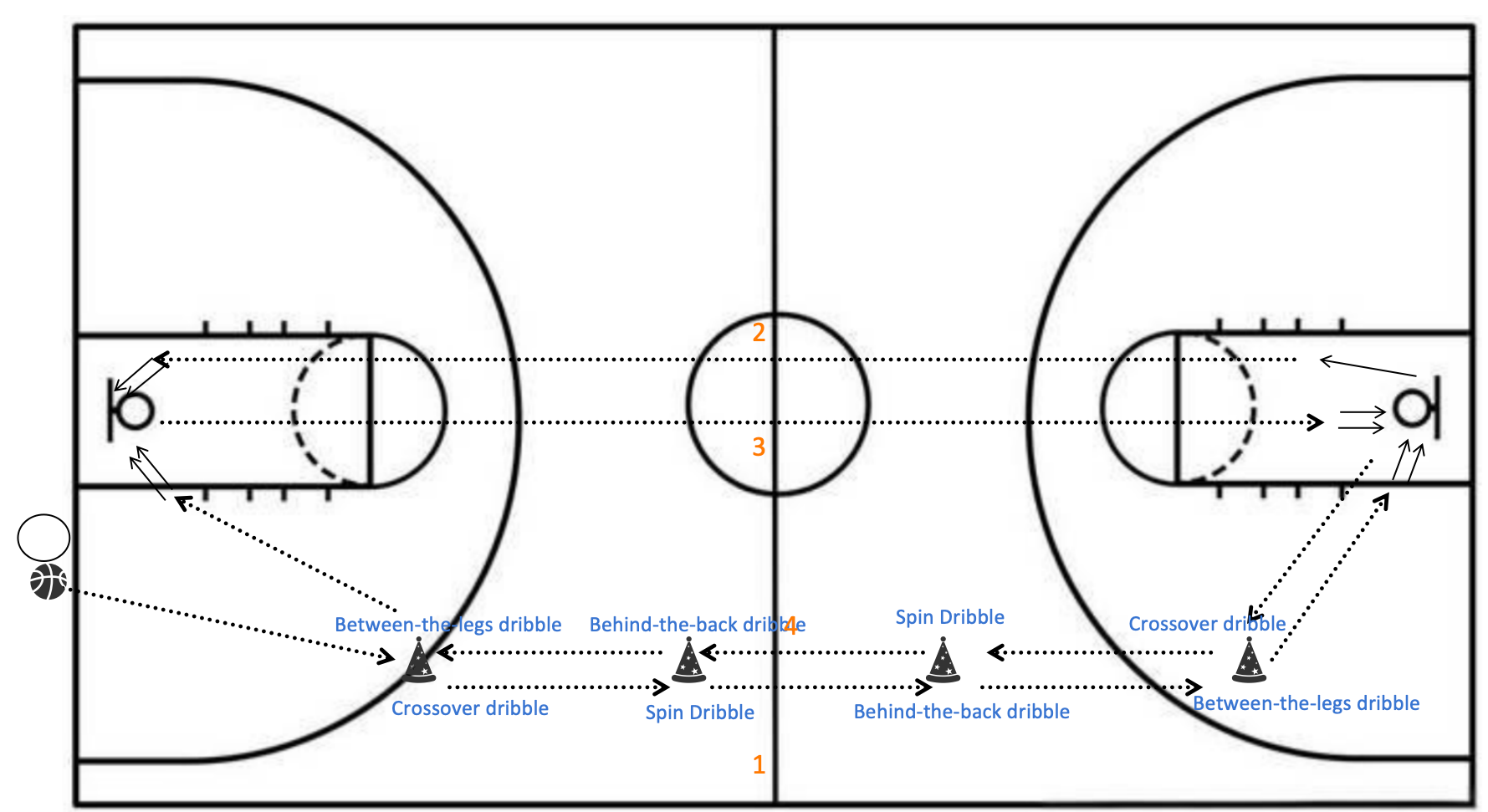 |
| 2.Multi-point passing/receiving circuit​ | Athletes initiate behind the baseline by throwing a two-hand overhead pass to Target 1. After releasing, they sprint to the free-throw line to receive a return pass from Target 1. Upon catching, execute a chest pass to Target 3 while relocating to half-court. Crossing midcourt, receive a pass from Target 3, perform a jump stop, then deliver a one-hand bounce pass to Target 2. Immediately close out to Target 2, receive a return pass with another jump stop, initiate a stridedrive penetration into the key, and finish with a one-hand pass to Target 5. Sprint to the opposite baseline, receive from Target 5 outside the endline, and fire a one-hand shoulder pass to Target 3. While relocating towards center court, receive from Target 3 near midcourt, then dribble across half-court line executing a one-hand tip pass to Target 1. Finally, sprint beyond the three-point line, catch with a two-step stride stop, and attempt a one-hand side/chest pass to strike the obstacle in the paint. Timing stops upon target impact or ball touchdown. 5 second penalty for missed obstacles. Total time recorded over two trials, best score retained.  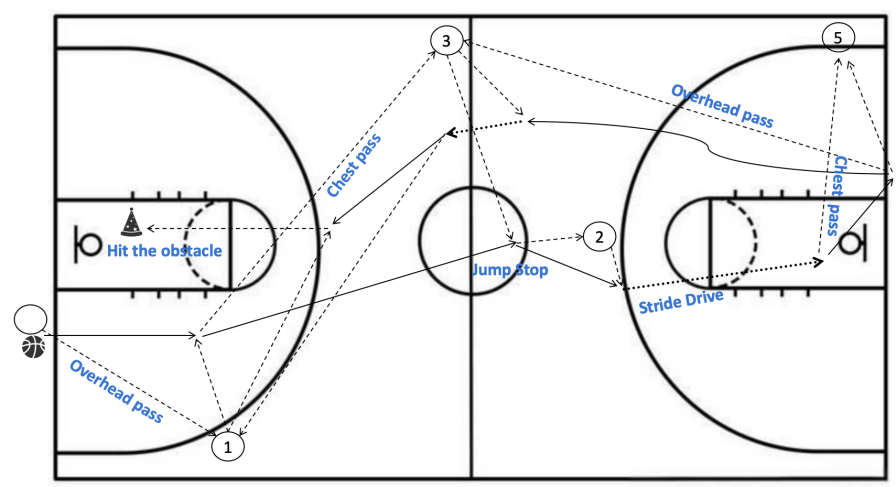 |
| 3.Hexagon shuffle drill | Athletes initiate from the central point, executing sequential movements to 6 markers positioned 5m equidistant per:  Sprint to Marker 1: Maximum acceleration→Two-foot jump stop→Light touch marker→Backpedal to center;  Crossover to Marker 2: Defensive crossover steps→Two-step stride stop→Touch→Crossover return;  Closeout to Marker 3: Offensive charge step→Touch→Right drop step→ Left drop step to center;  Lateral shuffle to Marker 4: Shuffle at shoulder-width stance→Touch → Shuffle return;  Slide-retreat to Marker 5: Forward slide→Touch→Backward slide return  Hybrid move to Marker 6: Crossover initiating→Transition to lateral shuffle→Touch→Hybrid return.  Total time recorded via laser timing gates,with two trials administered and best score retained. Penalties: +0.3s per missed touch.  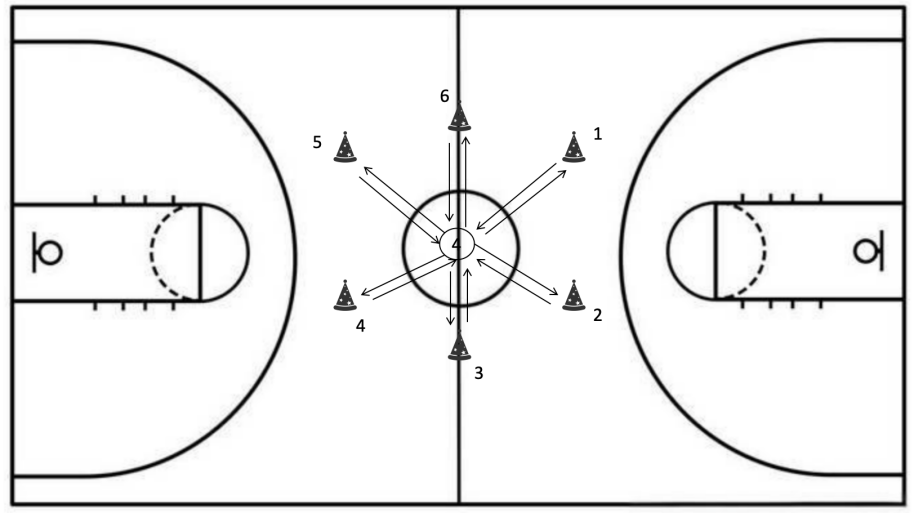 |
